# Oral Films Printed with Green Propolis Ethanolic Extract

**DOI:** 10.3390/polym16131811

**Published:** 2024-06-26

**Authors:** Leandro Neodini Remedio, Vitor Augusto dos Santos Garcia, Arina Lazaro Rochetti, Andresa Aparecida Berretta, Julieta Adriana Ferreira, Heidge Fukumasu, Fernanda Maria Vanin, Cristiana Maria Pedroso Yoshida, Rosemary Aparecida de Carvalho

**Affiliations:** 1 Faculty of Animal Science and Food Engineering, USP—University of São Paulo, Av. Duque de Caxias Norte 225, Pirassununga 13635-900, SP, Brazil; lneodini@utec.edu.pe (L.N.R.); arinarochetti@usp.br (A.L.R.); fukumasu@usp.br (H.F.); fernanda.vanin@usp.br (F.M.V.); 2 Faculty of Agricultural Sciences, UNESP—São Paulo State University, José Barbosa de Barros 1780, Botucatu 18610-034, SP, Brazil; vitor.as.garcia@unesp.br; 3 Research, Development & Innovation Department, Apis Flora Industrial e Comercial Ltda, Rua Triunfo 945, Ribeirão Preto 14020-670, SP, Brazil; andresa.berretta@apisflora.com.br; 4 FHO—Hermínio Ometto Foundation, Av. Doutor Maximiliano Baruto 500, Araras 13607-339, SP, Brazil; julieta.ferreira@fho.edu.br; 5 Institute of Environmental, Chemical and Pharmaceutical Sciences, UNIFESP—Federal University of São Paulo, Rua São Nicolau 210, Diadema 09913-030, SP, Brazil; cristiana.yoshida@unifesp.br

**Keywords:** tape casting, printing layers, roughness, stability

## Abstract

Oral film (OF) research has intensified due to the effortless administration and advantages related to absorption in systemic circulation. Chitosan is one of the polymers widely used in the production of OFs; however, studies evaluating the maintenance of the active principles’ activity are incipient. Propolis has been widely used as an active compound due to its different actions. Printing techniques to incorporate propolis in OFs prove to be efficient. The objective of the present study is to develop and characterize oral films based on chitosan and propolis using printing techniques and to evaluate the main activities of the extract incorporated into the polymeric matrix. The OFs were characterized in relation to the structure using scanning and atomic force electron microscopy; the mechanical properties, disintegration time, wettability, and stability of antioxidant activity were evaluated. The ethanolic extract of green propolis (GPEE) concentration influenced the properties of the OFs. The stability (phenolic compounds and antioxidant activity) was reduced in the first 20 days, and after this period, it remained constant.

## 1. Introduction

The feasibility of developing oral films as a delivery system for compounds with different biological activities has been studied, such as for the treatment of osteoarthritis [1], rosuvastatin bioavailability [2], hypertension [3], oral cancer [4], and anti-inflammatory activity [5], among others. Commercially, some pharmaceutical companies are already working with orodispersible films such as Sympazan^®^ (Otter Pharmaceuticals, Lake Forest, CA, USA), NiQuitine Strips^®^ (GlaxoSmithKline plc, London, UK), Gas-X Thin Strips^®^ (Novartis AG, Basel, Switzerland), and Listerine Pocket Packs^®^ (Johnson & Johnson, New Brunswick, NJ, USA). More recently, patients have been looking for complementary and integrative treatments or food supplementation based on natural products, which have been commonly used for centuries in folk medicine, and which have had their biological properties confirmed by non-clinical and clinical trials.

Propolis is produced by Apis mellifera bees; the physical and chemical aspects of propolis are used to protect their hives [6,7]. Propolis has demonstrated several biological properties in in vitro and in vivo models [7,8,9]. Of notable interest are its antimicrobial, antioxidant, anti-inflammatory, and immune regulatory effects [10,11,12,13]. Propolis was validated under some clinical trials for their antioxidant effects [14], kidney protection effects [15,16,17,18,19], and as a COVID-19 coadjutant therapy administered orally [20]; propolis has also been validated for its anti-Candida activity [21,22,23,24,25] and for its wound healing effects after topical application on the skin, mouth, and vaginal mucosa [10,26,27,28]. 

Alternatives for the administration of the natural active compound have been explored, such as gels [29,30,31,32], ointments [33,34,35], and mainly orally disintegrating films [1,5,36]. Generally, oral films are developed from natural polymers [1,3,36,37,38,39], and their characteristics, such as disintegration time, stability of the active compounds, and biological activities, among others, are determined since they can be affected by several factors.

The polymer is one factor that significantly affects oral films’ properties. Chitosan has recently been used in the production of oral films [40,41,42,43], and several studies have reported its advantages for its antimicrobial, anticancer [44,45,46], and film-forming capacity [40,41,47,48].

One of the limitations in oral film development is the concentration of drugs, and studies are needed to increase their incorporation capacity [49]. Bashir et al. reported that a disadvantage of oral films is that they generally do not offer dose flexibility [50]. Most of the research related to oral film development involves incorporating the active principle in the film-forming solution, which may imply the degradation of compounds, interaction of compounds with polymers (reducing the availability of the active principle), or even limitation of the viable concentration for incorporation. A relevant aspect is the hydrophilic character of the natural polymers used in the production of oral films, which could limit the characteristics of the active principles incorporated in the polymeric matrix. To be compatible with the natural polymer, the active compound should also have a hydrophilic character.

Printing techniques have recently been used to incorporate active compounds into oral films. They are considered simple, direct, and innovative, making it possible to print more than one layer, which could incorporate higher compound concentration. According to Dodoo et al., 2D printing or inkjet printing can reproduce digital image data on a substrate using ink droplets, which offers advantages such as high yield and reproducibility [51].

The incorporation of different compounds into polymeric matrices using printing techniques has been reported in the literature, such as theophylline anhydrous [52], triamcinolone acetonide [53], caffeine [54], ethanol pomegranate extract [55], and paracetamol [56]. 

Previous works involving the development and characterization of oral films incorporating an ethanolic extract of propolis using natural polymers such as gelatin [57], sodium alginate, agar, and chitosan [58] have already been performed. One of the main limitations was the incorporated dosage of the propolis ethanolic extract. More recently, Remedio et al. produced oral films based on HPMC with a green propolis standardized extract (EPP-AF^®^, Apis Flora Industrial e Comercial Ltda, Ribeirão Preto, Brazil) using a printing technique, which enabled the increase of the propolis concentration, according to the increase in the number of printing layers [5].

The objective of this study is to evaluate the potential of using chitosan to develop orally disintegrating films as carriers of a propolis ethanolic extract with printing techniques.

## 2. Materials and Methods

### 2.1. Materials

Chitosan, purchased from Polymar (degree of deacetylation = 89% [59], molar mass = 165.62 g/mol, Fortaleza, CE, Brazil), was used. Glacial acetic acid was purchased from LabSynth (Diadema, SP, Brazil) to adjust the pH of the filmogenic solution. The commercial ethanol extract of propolis (Green Propolis EPP-AF^®^, Apis Flora Industrial e Comercial Ltda, Ribeirão Preto, Brazil batch 144 008 19) was donated by Apis Flora Indl. Coml. Ltd.a, Ribeirão Preto, SP, Brazil), with the physical–chemical characteristics as described by Remedio et al. [5].

The chemical profile, evaluated using HPLC methodology, was previously published by Remedio et al. [5]. Of remarkable interest, it is possible to highlight the content of 3.741 ± 0.02 mg/g of drupanin, 5.191 ± 0.05 mg/g of Artepillin C, and 0.503 ± 0.01 mg/g of baccharin, which are characteristic compounds found in Brazilian green propolis.

### 2.2. Production of Oral Films (OFs)

Chitosan (CHI, constant concentration of 3 g of CHI/100 g of filmogenic solution—FS) was dispersed in an acetic acid solution. Stoichiometric acetic acid (LabSynth, São Paulo, Brazil) was added to produce the acid solution, and the volume of acid incorporated for CHI solubilization was calculated according to Yoshida et al. [60]. The CHI dispersion was kept under mechanical stirring (digital IKA RW20 shaker, 400 rpm, 2 h). Then, the filmogenic solution (FS) was kept in an ultrasonic bath (1 h, Ultra Clear—1400 A, Unique) overnight at room temperature (25 ± 2 °C). The FS was dispersed in acrylic plates using a Zehntner automatic film applicator (ZAA 2300—Zehntner, Zurich, Switzerland). The FS was spread with an adjustable film applicator wet coating film (thickness = 3000 µm). The OFs were dried at 40 °C (drying oven MA-035, Marconi) for 24 h.

A printing technique was used to incorporate green propolis ethanolic extract (GPEE). The OFs were printed using an Ecotank L396 Multifunctional printer (Epson, Seiko Epson Corporation, Suwa, Japan) with four ink tanks (black, yellow, magenta, and cyan). The four tanks were filled with GPEE. However, only the tank intended for the black printing solution was used [5]. The printing configuration was performed according to Borges et al. [55], which generated a black rectangle (15 cm × 22 cm) in the Microsoft Word program, and printing was configured with a “Premium Photo Paper Glossy” quality. The number of printed layers varied between 1 and 4 layers, and OFs without GPEE were used as a control. 

### 2.3. Characterization of Oral Films

#### 2.3.1. Visual Aspect and Color Parameters

The visual appearance of the films without GPEE was evaluated in relation to the formation of a continuous matrix, the presence of insoluble particles, and easy removal from the support. OFs with different numbers of printed layers were evaluated; the absence of a deformation of the film was due to the increase in the number of layers and the uniform distribution of GPEE in the OF area.

The a* (red/green coordinate), b* (yellow/blue coordinate.), and L* (lightness) values were determined using a colorimeter (Miniscan XE, HunterLab, Reston, VA, USA). To determine the color parameters, the OFs (5 cm × 5 cm) were superimposed on a white plate, and the readings were taken at five random points in their area to determine the arithmetic mean. 

#### 2.3.2. Surface pH

The surface pH of the OFs was determined using phosphate buffer (pH = 6.8 [61]) as a solvent, according to the methodology proposed by Prabhu et al. with modifications [62]. The OFs (3 cm × 2 cm) were placed (for 30 s) in a 0.5 mL buffer solution. A pH meter (WTW-3210, WTW Company, Weilheim, Germany) was used to determine the pH; the electrode was kept in contact with the OFs for 1 min. After this period, the pH was recorded.

#### 2.3.3. Disintegration Time (DT)

Disintegration time was determined according to the methodology proposed by Steiner et al. [63], with the apparatus described by Remedio et al. [5]. Water was used as a solvent. The OFs (3 cm × 4 cm) were fixed in the apparatus, and 0.9 mL of water at a temperature of 37 °C was deposited on the surface of the OFs; the ball was immediately deposited on top of the films. The DT disintegration time corresponds to the time needed for the ball to pass through the film in the apparatus.

#### 2.3.4. Mechanical Properties

The tensile strength and elongation at break were determined according to the ASTM D882-12 methodology [64], with the experimental test conditions set at 100 mm for the initial separation distance and the test speed at 1.0 mm/s. The analyses were performed using a TA.XT plus texturometer (probe A/TG tensile grips, TA Instruments, New Castle, DE, USA).

#### 2.3.5. Contact Angle

The contact angle was determined on an Attension Theta Lite tensiometer (KSV Instruments, Biolin Scientific AB, Gothenburg, Sweden) using deionized water as a solvent. The solvent (5 µL) was deposited on the surface of the OFs (2 cm × 3 cm) and the variation in the contact angle was determined as a function of the surface of the oral films (without and with GPEE printing) during a period of 300 s (image recording at intervals of 5 s).

#### 2.3.6. Scanning Electron Microscopy (SEM) and Atomic Force Microscopy (AFM)

The effect of incorporating GPEE using the printing technique on the structure of the OFs was evaluated using SEM and AFM. Before analysis, the OFs were kept in a desiccator containing silica gel (10 days, 25 ± 2 °C).

For the SEM analyses (Hitachi TM-3000 microscope, Hitachi, Tokyo, Japan), the OFs (10 mm × 10 mm) were fixed (carbon double-sided tape) directly onto the metallic sample holder. The operating conditions included 5 kv electron beams for the surface analyses and 15 kv for the internal structure (in this case, before the analyses, the OFs were fractured in liquid nitrogen).

The surface structure was analyzed using atomic force microscopy (atomic force microscope NT-MDT Solver Next, NT-MDT Spectrum Instruments, Moscow, Russia). Samples of OFs with and without GPEE printing (1 cm^2^) were fixed onto the equipment, and two-dimensional (2D) and three-dimensional (3D) images were captured. Image analysis was performed using the Image Analysis software (Image Analysis 3.2.5.12676).

#### 2.3.7. Fourier Transform Infrared Spectroscopy (FTIR)

The OFs (samples of 2 cm × 2 cm) were kept in desiccators containing silica gel (25 ± 2 °C) for 7 days to eliminate water. Possible interactions between GPEE and the chitosan-based polymer matrix were analyzed using infrared spectroscopy (FTIR, Pekin Elmer spectrophotometer with attenuated total reflectance (ATR) accessory, Spectrum One, New York, NY, USA). To obtain the spectra, 32 scans were carried out in the range of 650 to 4000 cm^−1^ (resolution of 4 cm^−1^).

Then, the FTIR spectra were mathematically treated, their baselines were corrected using the Savitsky–Golay filter, and then they were deconvoluted using the Gaussian function (R^2^ > 0.99, Origin 9.0.0 software from OriginLab, Originlab, Northampton, MA, USA). The absorption band at 1330 cm^−1^ (which could be attributed to the stretching vibrations of the C−N group of the chitosan) was used for the spectrum normalization of chitosan-based OFs without and with GPEE printing.

### 2.4. Stability

The stability of the active compounds in the OFs was monitored in relation to the concentration of total phenolic compounds and antioxidant activity (FRAP and ORAC methods) for a period of 90 days (intervals of 7 days in the first month and intervals of 15 days in the next 2 months). The storage conditions (58% relative humidity, 25 ± 2 °C and exposed to 150lux—Philips 100 W) of the OFs (samples 2 cm × 2 cm) were set as proposed by Borges et al. [55].

The extraction of active compounds from the active compounds of the OFs was carried out according to Remedio et al. [5], using 2 cm × 2 cm samples (approximately 0.1 g) with hydroalcoholic solution (80% ethyl alcohol). To determine the concentrations of phenolic compounds, the obtained extracts were diluted (1:1 for the control OFs, 1:5 for the OFs containing 1 and 2 printed layers of GEEP, and 1:10 for the OFs with 3 and 4 layers of GPEE).

Total phenolic compounds were determined according to Singleton et al. [65]. The absorbance determinations were performed at 740 nm using a Lambda 35 spectrophotometer (Perkin-Elmer, Waltham, MA, USA).

The FRAP method follows the methodology proposed by Benzie and Strain [66]. The analyses were carried out at 593 nm (Perkin-Elmer Lambda 35 spectrophotometer, Waltham, MA, USA); a curve was used for external calibration using Trolox as standard (2.5 and 22.5 µmol of Trolox/L and ethanol 80% as blank).

Determinations of antioxidant activity using the ORAC method were performed according to Ou et al. [67], using a spectrofluorimeter (BMG Labtech, FLUOstar OPTIMA, Offenburg, Germany; excitation wavelength = 485 nm and emission wavelength = 528 nm) and microplates (96 cells, Greiner Bio-One, Frickenhausen, Germany). A fluorescein solution (Sigma-Aldrich, Saint Louis, MO, USA, 81 nM) was used, and the samples were incubated for 10 min at a temperature of 37° C. After this period, AAPH (2,2′-Azobis(2-methylpropionamidine) dihydrochloride; 25 µL; 152 mM) was used. The fluorescence decay was analyzed over 120 min (1 min intervals). Trolox was used as an external standard (12 to 96 µmol of Trolox/L and ethanol 80% as blank).

### 2.5. Statistical Analysis

The different formulations of OFs (without GPEE printing and OFs with 1 to 4 layers of GPEE printing) were produced at three different times (triplicate), with nine samples for each formulation, except for the mechanical properties, where ten analyses were performed for each triplicate (totaling thirty samples). The difference between means (analysis of variance, ANOVA) was analyzed using InfoStat software (Version 5.13.1, Tukey test with 95% confidence). 

## 3. Results and Discussion

### 3.1. Visual Appearance and Color Parameters

**Figure 1:**
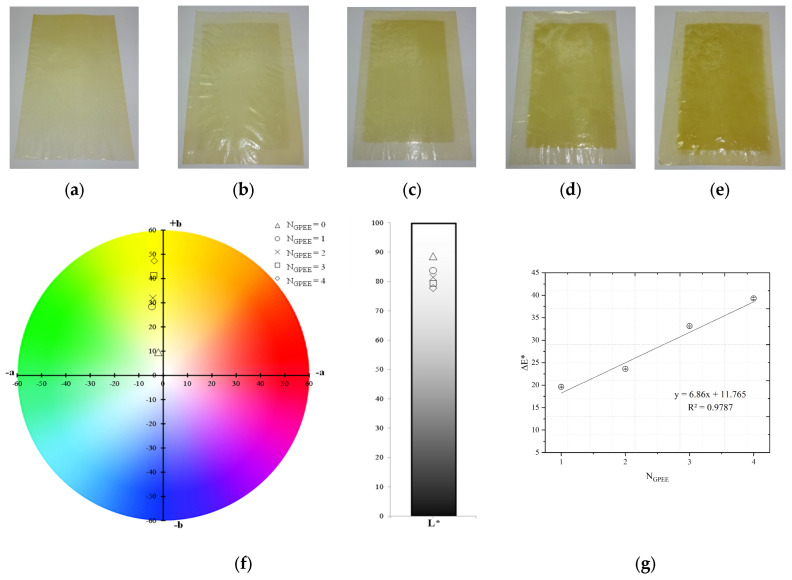
Chitosan-based oral films with and without printing of green propolis ethanolic extract (GPEE): (**a**) without printing (control); (**b**) number of print layers = 1; (**c**) number of print layers = 2; (**d**) number of print layers = 3 (**e**) number of print layers = 4; (**f**) color parameters (chroma a*, chroma b* and luminosity); and (**g**) color difference (ΔE*). Note: NGPEE = number of GPEE-printed layers.

Chitosan is one of the most used polymers to produce oral films in the literature [40,41,42,68], mainly due to its excellent ability to form films with or without the incorporation of plasticizers. The control film (Figure 1a, only chitosan) exhibited the formation of a continuous matrix and the absence of insoluble particles. Similar characteristics were observed for oral films printed with GPEE (Figure 1b–e). Additionally, the increased number of printed layers caused small deformations (which did not make production unfeasible). The GPEE distribution was visually uniform in the OF area, regardless of the number of printed layers. The results may be related to the reduced viscosity and surface tension of the GPEE. It was also verified that after the fifth printed layer, the OFs presented extremely malleable and deformed characteristics (due to the excess of printing solution and, consequently, the wet surface of the OFs), making it unfeasible to print with more layers.

In oral films based on carboxymethyl cellulose (CMC), printing one to four layers of ethanol pomegranate extract did not interfere with film formation [55]. The same results were observed in CMC-based orally disintegrating films (one to four layers) using GPEE as a printing solution [5]. It was possible to print oral films with nine layers in HPMC-based films using rasagiline mesylate as an active pharmaceutical ingredient (API) [69].

Regarding the color parameters (Figure 1f), significant changes were observed in the parameters a* and b* between the OFs with and without GPEE printing. The increase in the number of printed layers caused an increase in the a* and b* values, indicating an increase in the intensity of the yellow color with the increase in the number of printed GPEE layers (Figure 1g). It was verified that the increase in the number of layers caused a similar reduction in L* in relation to the OFs without the incorporation of GPEE, indicating a darkening due to the increase in the concentration of GPEE on the surface of the OFs.

The increases in the number of layers caused a linear increase in the total color difference (ΔE*) in relation to the control OFs (without GPEE printing) (Figure 1g). The results of the color parameters indicated an increase in the concentration of the extract in the polymeric matrix, which may be interesting for controlling the dosage in terms of the desired properties.

### 3.2. Surface pH

The surface pH values with or without the incorporation of GPEE (Table 1) were in the range reported in several works in the literature that do not present a great risk for oral mucosa [70,71]. However, the increase in impression layers has caused a significant reduction in pH values. The pH reduction is related to the pH of the GPEE (pH = 5), as reported by Remedio et al. [5]. The surface pH depends on the OF formulation (polymer, active ingredient, plasticizers, solvents used in the solubilization of the polymer, and the active ingredient, additives, among others). Additionally, there is no standardization of the solvent used in pH determination; in some studies, water is used as a solvent, and in others, a saliva-simulating solution (phosphate buffer) is used, among others. As a result, the comparison with other chitosan-based OFs is complex.

The pH surface values between 3.60 and 5.53 were verified in studies related to the production of mucoadhesive buccal films (with and without incorporation of active principle) based on different types of chitosan and solvents, using water as a solution, such as ChitopharmTM—98% degree of deacetylation, ChitopharmTM—89% degree of deacetylation, ChitopharmTM—90% degree of deacetylation na ViscosanTM—50% degree of deacetylation [68]. Khajuria et al. verified pH values of 6.5 (regardless of the type of chitosan) for films based on chitosan and metformin using a McIlavine buffer (pH = 6.6) as a solvent [72]. The values were similar to those observed by Remedio et al. for orally disintegrating films based on HPMC (values between 5.74 and 6.85) and printed with the same extract (1 to 4 printed layers) [5].

### 3.3. Disintegration Time (DT)

The OFs with the incorporation of GPEE (regardless of the number of layers) presented higher values of disintegration time (Table 1) in relation to the control (without impression). There were no significant differences in the disintegration time between the OFs with different numbers of printed layers. Korelc et al. developed oromucosal films prepared using a solvent-cast-evaporation method from different types of chitosan (different molecular weights, degree of deacetylation, pattern of deacetylation) containing prednisolone [68]. They found that regardless of the type of chitosan used, the oromucosal films did not disintegrate after 3 h of exposure in simulated saliva at 37 °C.

The DT results differed from those observed for films based on hydroxypropyl methylcellulose, where the OFs with different numbers of GPEE-printed layers did not show significant differences in the control OFs [5]. The DT values (>300 s) were higher than those reported in the literature for orally disintegrating films. According to Jyoti et al., oral films that present a disintegration time of a maximum of 60 s are classified as fast; in a few minutes, these oral films release a mucoadhesive gel. Oral films that present a disintegration time of a maximum of 8–10 h are classified as films with a controlled mucoadhesive release [73]. Therefore, the chitosan-based oral films with different GPEE-incorporated layers can be classified as mucoadhesive release oral films.

Chitosan is stable under neutral conditions due to the strong hydrogen bonds between the amine and hydroxyl groups, which crystallize [74]. In this work, the interaction of these sites may have contributed to the high DTs observed. There are several studies reported in the literature in which chitosan is used to produce oral mucoadhesive films [75,76], as it has numerous groups that form hydrogen bonds with chains of polymeric substances that can penetrate mucus and epithelial tissue [50], which can contribute to the high DTs observed.

### 3.4. Mechanical Properties

**Figure 2:**
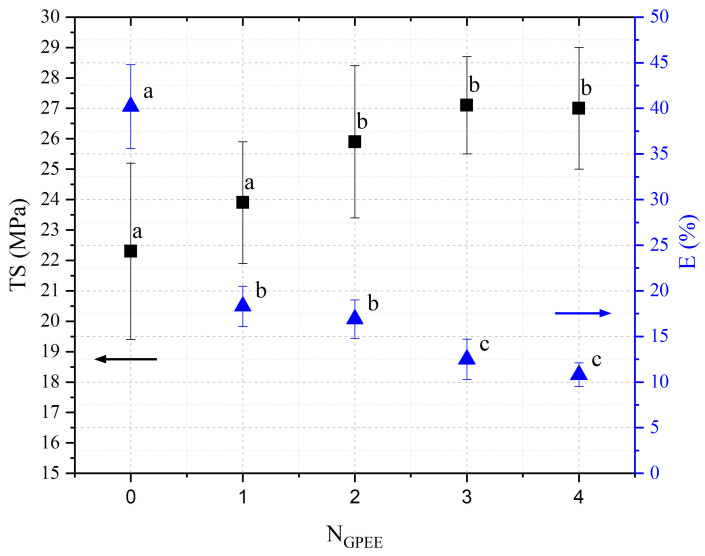
Tensile strength (TS) and elongation (E) of chitosan-based oral films with and without the printing of green propolis ethanolic extract (GPEE). Note: NGPEE = number of GPEE-printed layers. Different letters indicate significant differences between the means (Tukey’s test, *p* < 0.05).

The thickness of the chitosan-based OFs varied between 0.078 mm (control, OFs, without printing) and 0.079 mm (OFs with different numbers of GPEE-printed layers). There were no significant differences between the thickness of control OFs and those printed with GPEE, regardless of the number of printed layers. The results may be associated with the absorption of the extract in the polymeric matrix during the printing process. The thickness results corroborate what was observed in the atomic force microscopy, where a reduction in roughness was observed on the surface of the OFs (Figure 2).

The printed OFs showed higher tensile strength (Figure 2) compared to the control OFs (no printing), with no significant differences being observed (*p* < 0.05) with the increase in the number of printed layers (two to four layers). However, elongation values were reduced (Figure 2), increasing the number of printed layers. The results are possibly associated with the GPEE penetration and deposition that modify the molecular mobility and, consequently, the mechanical properties. According to Remedio et al., using GPEE (the same used in this work) as a printing solution can cause structure filling in orally disintegrating films [5].

Li et al. [77] produced oral disintegration films based on pre-gelatinized waxy corn starch and different concentrations of chitosan (10 to 30%) and reported that compared to the control oral disintegration film (pre-gelatinized waxy corn starch), the tensile strength and elongation of the films increased by up to 20% (*w*/*w*), presenting values between 2 to 5 MPa and 20 to 40% for TS and E, respectively. The authors attributed this effect to the formation of more uniform chains after the addition of chitosan in the oral disintegration films. 

The mechanical strength is important in all production stages until consumption by the patient to guarantee a product that does not cause physical harm; thus, similar mechanical properties were obtained at the time of consumption to those obtained after production [78]. In this context, there are no reference values for the mechanical properties of oral disintegrating films.

### 3.5. Contact Angle

**Figure 3:**
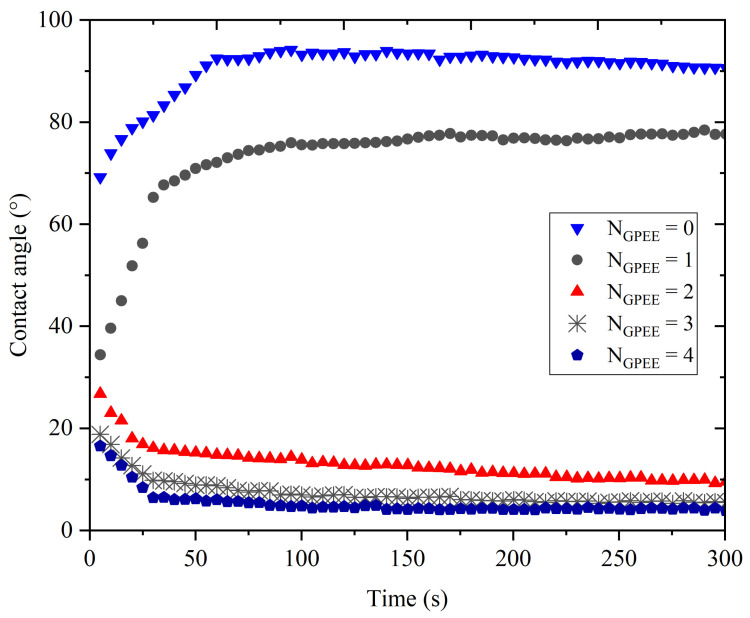
Effect of time on the contact angle of oral films based on chitosan-based oral films with and without printing of green propolis ethanolic extract (GPEE): (▼) without printing (control); (●) number of print layers = 1; (▲) number of print layers = 2; (𐠀) number of print layers = 3 (⬟) number of print layers = 4.

The variation in the contact angle as a function of time (Figure 3) indicated that the increase in the number of printed layers promoted a reduction in the contact angle in relation to the control OF (without GPEE printing). The contact angle values of chitosan-based films were greater than 90 °C, indicating a hydrophobic character [79,80]. However, the incorporation of GPEE caused a reduction in the contact angle, indicating greater surface hydrophilicity. The angle reduction may be associated with physical factors and perhaps reduced roughness due to the deposition of the extract on the surface using the printing technique. On the other hand, the literature reports that the hydrophilicity reduction is associated with the presence of hydroxyl groups of flavonoids in the ethanolic extract of propolis [81].

An anomalous behavior was verified for the control films (without printing) and the films with a layer of GPEE; evaluating the variation in the contact angle with the deposition time, an increase in the angle of contact in the first 60 s, followed by stability in both cases, was observed. The initial swelling of the polymeric matrix may have caused an increase in the contact angle, indicating a hydrophobic characteristic of the control OFs (without impression) and the films with one layer of GPEE. There was a drastic reduction in the contact angle of OFs with two to four printed layers in relation to the OFs without printing and with one printed layer and their stabilization after this period. It is possible that after the first printed layer, there is a greater absorption of the extract (observed by the reduced roughness of the OFs, Figure 3), which consequently favors the spreading and absorption of the decrease on the surface.

### 3.6. Scanning Electron Microscopy (SEM) and Atomic Force Microscopy (AFM)

The analyses of the surface of the OFs using SEM (Table 2) showed that the control OF presented a homogeneous structure. However, there were marks on the surface related to the printing process (however, the marks were not visually observed for the OFs that incorporated GPEE, regardless of the number of printed layers). In the internal structure (Table 2), there were reduced particle sizes in the OFs with and without GPEE layers, possibly signifying insoluble chitosan particles.

AFM can verify more relevant observations regarding extract absorption. The micrographs obtained using AFM (Table 2) indicate that the OFs without GPEE present a higher roughness. Increasing the number of GPEE layers causes a reduction in the roughness of the OFs. The average roughness values (AR, Table 2) determined can confirm the results. In the case of chitosan-based OFs, apparently, there is a partial absorption of GPEE (which may be related to porosity and interactions of GPEE with chitosan) after printing the first layer and increasing the number of printed layers, causing the filling of the voids in the polymeric matrix and forming a film on the surface of the OFs.

Borges et al. analyzed the effect of incorporating different concentrations of pomegranate extract using the printing technique on CMC-based orally disintegrating films [55]. They observed a similar effect, where the increase in the number of layers added caused a decrease in the roughness of the film. According to the authors, these results may be related to the absorption of the extract in the polymeric matrix with increases in the printing layers.

### 3.7. FTIR

**Figure 4:**
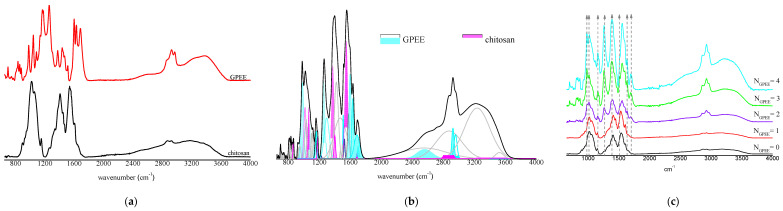
(**a**) Fourier transform infrared absorbance spectra of chitosan-based oral films and GPEE; (**b**) analytical deconvolution curves of chitosan-based oral films with four printed layers of GPEE (some deconvoluted analytical curves of GPEE and chitosan bands are highlighted in different colors under the FTIR absorption spectrum); (**c**) FTIR absorption spectra of chitosan-based oral films with and without green propolis ethanolic extract (GPEE) printing. Note: NGPEE = number of GPEE-printed layers.

The FTIR absorbance spectra for chitosan-based OFs and GPEE are shown in Figure 4a. It is possible to identify characteristic absorption bands of chitosan-based OFs: an overlapped wide absorption band located between 3600 and 3000 cm^−1^ is due to the stretching vibration of hydroxyl groups (−OH) and amino groups (−NH); bands at 2928 and 2870 cm^−1^ correspond to CH− symmetric and asymmetric stretching; bands with a maximum of 1652 cm^−1^ are due to C=O stretching, and of 1330 cm^−1^, attributed to CN− stretching; two absorption bands with a maximum of 1410 and 1381 cm^−1^ relate to −CH2 bending and −CH3 symmetrical deformations, respectively; an absorption band at 1152 cm^−1^ corresponds to the asymmetric stretching of the C−O−C bond; bands at 1062 and 1019 cm^−1^ are attributed to CO− stretching; and the absorption band at 896 cm^−1^ corresponds to −CH bending [82].

It is possible to observe typical absorption bands for GPEE: an overlapped wide absorption band with a maximum at 3886 and 3250 cm^−1^ is related to −OH groups from the phenolic compounds of propolis and the ethanol used for GPEE; bands between 2969 and 2850 cm^−1^ are due to −CH2 symmetrical and asymmetric stretching, respectively; a band with a maximum at 1683 cm^−1^ is attributed to C=O stretching; peaks at 1600 and 1513 cm^−1^ are due to C=C−C aromatic ring stretching vibrations; an absorption band located at 1465 cm^−1^ is related to the stretching vibration of C=C groups; absorption bands with a maximum at 1437 and 1375 cm^−1^ are attributed to −CH3 asymmetrical bending and to −CH3 symmetrical bending vibrations, respectively; a peak at 1261 cm^−1^ is attributed to −OH group in-plane bending; an absorption band at 1168 cm^−1^ is due to C−O stretching vibrations of tertiary alcohols; a peak at 1044 cm^−1^ is due to the −CO stretching vibration of primary alcohol [83]. 

Figure 4b presents an example of the analytical deconvolution curves obtained from the FTIR absorbance spectrum of chitosan-based OFs with four GPEE-printed layers. This procedure was also performed in chitosan-based OFs without GPEE and with one, two, and three GPEE-printed layers.

The FTIR absorbance spectra of the OFs with one and two printed layers are quite similar to the control OFs. The appearance of new absorption bands was observed for the OFs with three and four printed layers. In general, the intensity of some absorption bands is higher for OFs with four printed layers. These absorption peaks are attributed to the stretching and bending vibrations of the GPEE, such as at 2969 and 2850 cm^−1^, due to –CH2 symmetrical and asymmetric stretching, respectively; at 1683 cm^−1^, referring to C=O stretching vibration; and at 1600 and 1513 cm^−1^, due to C=C−C aromatic ring stretching vibrations. The stretching of C=O, C−C=C, and C−O groups in the phenolic components can be observed between 1800 and 1000 cm^−1^ [84].

Figure 4c shows the FTIR absorption spectra of chitosan-based OFs with and without printed layers of GPEE. From the analytical deconvolution curves, it was possible to normalize the absorption spectra using the absorption band at 1330 cm^−1^, which is attributed to the stretching vibrations of the C−N group of the OFs [84,85]. There was only an increase in the intensity of the absorption bands, which was attributed to the printed GPEE. No shift in the wavelengths of the absorption bands was observed. This suggests that the GPEE is physically retained on the OFs, which is as expected when using the printing process.

### 3.8. Stability 

**Figure 5:**
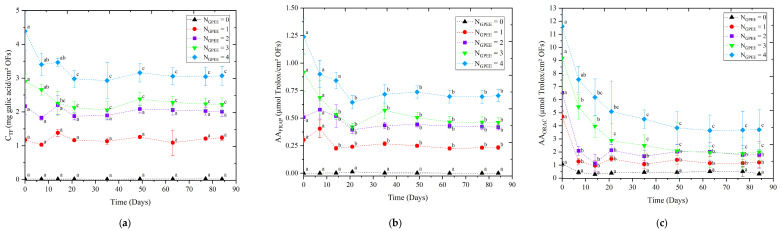
Stability over time of chitosan-based oral films with and without printing of green propolis ethanolic extract (GPEE): (**a**) concentration of total phenolic compounds (CTF); (**b**) antioxidant activity determined using the method of FRAP (AAFRAP) and (**c**) antioxidant activity determined using the oxygen radical absorbance capacity (AAORAC). Note: NGPEE = number of GPEE-printed layers. Different letters indicate significant differences between the means (Tukey’s test, *p* < 0.05).

It was verified that the increase in the number of printed layers caused an increase in the concentration of phenolic compounds (Figure 5a), as expected, due to the increase in the concentration of GPEE. The purpose of printing different numbers of layers was to control the concentration of the active ingredients. Similar results, namely an increased concentration of phenolic compounds, were observed by Remedio et al. for HPMC-based oral disintegration films and the printing of propolis ethanolic extract [5]. Variations in the concentration of phenolic compounds were verified in the first 20 days. After this period, no significant variations were observed. Borges et al. produced films with lecithin, gelatin, and different concentrations of hydrolyzed collagen and propolis ethanolic extract and reported values of total phenolic compounds in the range of 41.5 to 63.0 mg of gallic acid/g of dry film [86]. 

A similar behavior was observed in relation to the antioxidant activity. The increases in the number of printed layers caused an increase in the antioxidant activity (Figure 5b,c). In the first 20 days of storage, a significant reduction (*p* < 0.05) of antioxidant activity was observed. After this period, no significant differences were observed. The results indicated that the use of this printing technique is interesting for the incorporation of active principles in oral films, observing concentrations of phenolic compounds, and antioxidant activity. Yu et al. reported that an oral mucosal adhesive film based on HPMC and curcumin (core material), which was produced using the 3D printing method, showed antioxidant activity and that the increase in core material led to a proportional increase in the antioxidant activity of oral films (assessed by DPPH radical method), which remained stable for 15 days [87]. 

The printing technique was noteworthy for the incorporation of the active principle of the compound in oral films; concentrations of phenolic compounds and antioxidant activity were observed.

## 4. Conclusions

The results indicated that printing techniques can be used for incorporating active compounds from natural sources into chitosan-based oral films, with the limiting factor being the number of printed layers. Overall, the number of printed layers affected the properties of the oral films. The FTIR results indicated that there was no interaction between the ethanolic extract of propolis and the polymer used, indicating that the incorporated concentration will effectively be available. Additionally, significant concentrations of phenolic compounds and antioxidative potential were found in the oral films for a period of 90 days. 

## Figures and Tables

**Table 1:** Surface pH and disintegration time of chitosan-based oral films (OFs) with and without printing with different numbers of layers of green propolis ethanolic extract (GPEE).

Printing Layers	Surface pH	Disintegration Time (s)
0	6.91 ± 0.02 ^a^	331 ± 129 ^a^
1	6.54 ± 0.21 ^b^	454 ± 155 ^a,b^
2	6.36 ± 0.17 ^c^	312 ± 243 ^a^
3	6.02 ± 0.09 ^d^	532 ± 166 ^b^
4	5.89 ± 0.11 ^e^	557 ± 22 ^b^

Different letters indicate a significant difference (*p* < 0.05) between means using the Tukey test.

**Table 2:** Micrograph of the surface (MEV surface) and internal structure (MEV internal), atomic force microscopy images of the surface in 2D (AFM 2D) and 3D (AFM 2D), and average roughness (AR) of chitosan-based oral films without (printing layers = 0) and with printing of green propolis ethanolic extract (printing layers = 1, 2, 3, and 4).

Analyses	Printing Layers of Green Propolis Ethanolic Extract				
	0	1	2	3	4
MEV Surface	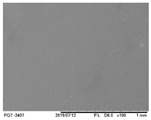	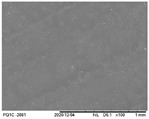	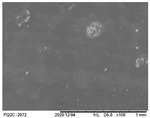	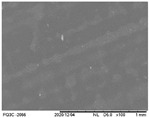	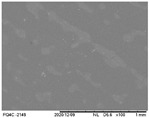
MEV Internal	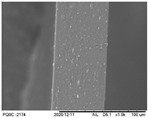	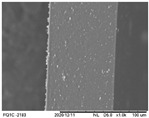	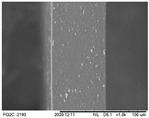	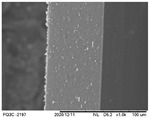	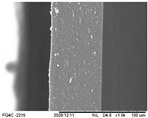
AFM 2D	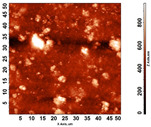	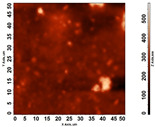	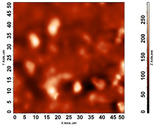	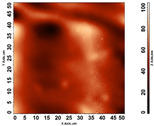	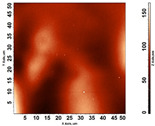
AFM 3D	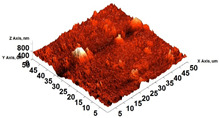	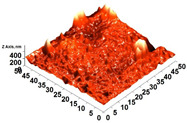	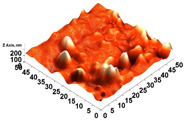	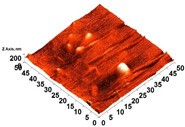	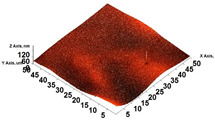
AR	81.95 ± 12.76 ^a^	50.18 ± 8.07 ^b^	37.87 ± 9.38 ^b^	32.33 ± 8.69 ^b^	12.37 ± 6.10 ^c^

Different letters indicate a significant difference (*p* < 0.05) between means using the Tukey test.

## Data Availability

The raw data supporting the conclusions of this article will be made available by the authors on request.
